# Humic Acid Derived from Vermicompost Improves Bone Mineral Content and Alters Oxidative Stress Markers in Ovariectomized Mice

**DOI:** 10.3390/biomedicines13020495

**Published:** 2025-02-17

**Authors:** Thays Cristina dos Santos, Hellen Paulo Silva, Karen Rodrigues Lima, Maria Luiza Nonato Salvador, Geraldo de Sousa Cândido, Laura Cristina Jardim Pôrto Pimenta, Natália Oliveira Bertolini, Luciana Botelho Ribeiro, Filipe Gomes Fagundes, Débora Ribeiro Orlando, Bruno Del Bianco Borges, Marco Fabrício Dias-Peixoto, Alan Rodrigues Teixeira Machado, Leonardo Barros Dobbss, Luciano José Pereira, Eric Francelino Andrade

**Affiliations:** 1 Faculty of Health Sciences, Universidade Federal de Lavras (UFLA), Lavras 37200-000, Minas Gerais, Brazil; thays.santos1@estudante.ufla.br (T.C.d.S.); hellen.silva7@estudante.ufla.br (H.P.S.); karen.lima1@estudante.ufla.br (K.R.L.); maria.salvador@estudante.ufla.br (M.L.N.S.); geraldo.candido@ufla.br (G.d.S.C.); laurap@ufla.br (L.C.J.P.P.); lulu_bribeiro@hotmail.com (L.B.R.); debora.orlando@ufla.br (D.R.O.); bruno.borges@ufla.br (B.D.B.B.); lucianojosepereira@ufla.br (L.J.P.); 2 Department of Physical Education, University Center of Lavras (UNILAVRAS), Lavras 37200-000, Minas Gerais, Brazil; natioliveira.ef@gmail.com; 3 Department of Exact Sciences, Universidade do Estado de Minas Gerais, João Monlevade 35930-314, Minas Gerais, Brazil; fgfagundes@gmail.com (F.G.F.); alan.machado@uemg.br (A.R.T.M.); 4 Postgraduate Program in Health Sciences (PPGCS), Federal University of the Jequitinhonha and Mucuri Valleys (UFVJM), Diamantina 39803-371, Minas Gerais, Brazil; marcofabri@ufvjm.edu.br; 5 Institute of Agrarian Sciences, Universidade Federal dos Vales do Jequitinhonha e Mucuri (UFVJM), Unaí 38610-000, Minas Gerais, Brazil; leonardo.dobbss@ufvjm.edu.br

**Keywords:** menopause, estrogen depletion, humic substances, bone, natural products

## Abstract

**Background:** Estrogen depletion alters bone mineralization and oxidative stress. Antioxidants like humic acids (HA) may help mitigate bone demineralization and redox imbalances. Thus, this study evaluated the effects of HA on bone mineral composition and oxidative stress markers in an experimental menopause model. **Methods:** Twenty-four female C57BL/6 mice were divided into four groups (n = 6/group): Sham; Sham + HA; Ovariectomized (OVX); and OVX + HA. The menopause model was induced by bilateral ovariectomy at the beginning of the experiment. HA derived from biomass vermicompost was administered daily by gavage for 28 days. After euthanasia, femurs and fragments of the gastrocnemius muscle, liver, and kidney were collected. Bone elemental composition was analyzed using scanning electron microscopy (SEM) coupled with energy dispersive spectroscopy (EDS). Superoxide dismutase (SOD), catalase (CAT), and hydrogen peroxide (H_2_O_2_) activities were assessed in muscle, renal, and hepatic tissues. Data were analyzed using two-way ANOVA and Bonferroni’s post hoc test. **Results:** Untreated OVX mice exhibited a significant reduction in femoral calcium content (*p* < 0.05). However, HA treatment increased calcium levels and improved the Ca/P ratio (*p* < 0.05). H_2_O_2_ activity was reduced in the liver and kidney of OVX + HA mice compared to untreated animals (*p* < 0.05). CAT activity in muscle increased in the OVX + HA group compared to the OVX (*p* < 0.05). **Conclusions:** HA treatment improved femoral elemental composition and modulated oxidative stress markers in an experimental menopause model.

## 1. Introduction

The climacteric is characterized by the gradual depletion of ovarian follicles, resulting in a reduction in estrogen production and ultimately leading to menopause [1]. While this phase is a natural transition in the female organism, it increases the risk of various health conditions, including coronary heart disease, Alzheimer’s disease, metabolic disorders, and osteoporosis [2]. Menopause-related osteoporosis is defined by the deterioration of bone microarchitecture and a reduction in bone mass, which leads to increased fragility and a higher susceptibility to fractures [3]. Moreover, estrogen deficiency during menopause increases oxidative stress, characterized by an imbalance between the production of reactive oxygen species (ROS) and the cell’s antioxidant defense mechanisms [4].

Due to hormonal factors, women with estrogen reduction are 1.5 times more likely to develop osteopenia/osteoporosis [5,6]. It is estimated that one in three women over the age of 50 will experience fractures related to this condition [5,6]. In 2019, the global incidence of osteoporosis was estimated at 41.5 million cases, with projections suggesting that more than 263.2 million people will be affected by the disease between 2030 and 2034 [7]. Additionally, the prevalence of osteoporosis is notably higher in low-income countries, where the substantial cost of treatment poses a significant challenge to public health systems [5,8].

Estrogen hormone therapy has been utilized in the treatment of menopausal patients to mitigate bone alterations caused by estrogen deficiency [9]. However, this form of replacement therapy has been associated with an increased risk of certain types of cancer, particularly breast and endometrial cancer [10,11]. Consequently, the search for novel therapies with fewer side effects and greater efficacy for preventing or treating menopause-related outcomes remains a significant area of interest [12,13]. It is considered that antioxidant agents can regulate bone remodeling and mitigate the loss of bone mineral density in osteoporosis [14]. Additionally, natural products with antioxidant activity may reduce oxidative damage and enhance the activity of antioxidant enzymes in metabolically active tissues [15,16]. Accordingly, humic acids (HA) are among the compounds highlighted for their potential to attenuate bone resorption [17].

HA are macromolecules primarily found in soils, rivers, oceans, and coal [18]. These compounds are naturally formed through chemical and physical reactions involving microbial residues and plant decomposition [19]. Additionally, HA can also be obtained through vermicomposting, a process in which organic matter undergoes aerobic decomposition facilitated by earthworms [20]. This process potentially enhances the biological activity of the resulting material [21]. Due to their antibacterial, anti-inflammatory, and antioxidant properties [22], the effects of HA have been investigated in both animal and human health, yielding promising results [17,23,24]. The administration of HA in experimental models has demonstrated their potential as wound-healing agents [25]. Additionally, these compounds have shown beneficial effects in the treatment of colitis, attributed to their role in modulating the gut microbiota [26], as well as an ability to improve the inflammatory profile in periodontitis models [27,28,29].

In recent studies conducted by our group, we observed that animals with ligature-induced periodontitis treated with HAs showed improvements in the mineral composition of alveolar bone and, consequently, exhibited reduced alveolar bone loss [17,29]. Given that both periodontitis and osteoporosis share similar mechanisms that promote bone resorption [30,31], it is plausible that agents capable of attenuating alveolar bone loss in periodontitis could also yield improvements in bone tissue affected by osteopenia/osteoporosis. To date, no studies have evaluated the effects of HA treatment on conditions associated with estrogen deficiency, such as osteopenia/osteoporosis. Therefore, we aimed to investigate the effects of HA treatment on bone elemental composition and oxidative stress parameters in an experimental model of ovariectomy-induced bone loss.

## 2. Materials and Methods

This study was approved by the Animal Use Ethics Committee of the Federal University of Lavras (CEUA/UFLA—protocol 3643011123). All procedures were conducted following the Guide for the Care and Use of Laboratory Animals and ARRIVE guidelines. The in vivo study was conducted between August and September of 2024.

### 2.1. Composting, Vermicomposting, Extraction, and Characterization of Humic Acids (HA)

Residues from soybean (*Glycine max* L.) and sorghum (*Sorghum bicolor* (L.) Moench), coarsely chopped, as well as corn grain flour (*Zea mays* L.) and crushed sugarcane bagasse (*Saccharum officinarum* L.) were composted for 30 days. The process included mechanical turning every 10 days. Subsequently, earthworms (*Eisenia foetida*) were added at a density of 50 per kilogram of organic material to initiate the composting process, which lasted approximately three months (95 days). Humidity, temperature, pH, and aeration parameters were monitored every three days. At the end of the process, the vermicompost was dried in an oven at 60 °C for 48 h, sieved through a 4 mm mesh, and used for the extraction of HA.

The extraction process began by treating the vermicompost with a 0.1 mol L^−1^ NaOH solution in a ratio of 1:10 (m/v) for four hours under agitation. After this period, the material was centrifuged (15 min at 5000 g) to separate the humic substances (humic acids − HAs + fulvic acids − FAs) from the humins (the insoluble fraction of organic matter). To separate the HAs from the FAs, the pH of the humic substances was reduced to between 1.0 and 1.5 by adding 6 mol L^−1^ HCl. At this pH, the HAs precipitated and were subsequently separated and washed with distilled water until a negative test for the presence of chloride was confirmed using silver nitrate (AgNO_3_). After washing, the HAs were titrated to a pH of 7.0 using 0.01 mol L^−1^ KOH and then placed in membranes with a molecular weight cut-off of 1000 Da and dialyzed against distilled water until the electrical conductivity (EC) of the system reached equilibrium. Following dialysis, the HAs were frozen and lyophilized for later use. The characterization of the HAs used in the present study has been described in previous studies conducted by our group [17,29,32].

### 2.2. Animals

Twenty-four healthy female C57BL/6 mice, aged 12 weeks and with an initial body weight of 20.8 ± 0.8 g, were used in this study. The animals were obtained from the NUCAL—Laboratory Animal Breeding Center (Universidade Federal de São João del Rey, Minas Gerais, Brazil). Throughout the experiment, the mice were housed in an experimental room under controlled conditions, including a temperature of 22 ± 2 °C, humidity of 45 ± 15%, and a 12 h light/dark cycle. Rodents were kept in polyethylene cages lined with wood shavings and provided with rodent chow (Nuvilab^®^) and water ad libitum for the entire experimental period.

### 2.3. Experimental Design

We performed a sample size calculation to ensure that the minimum number of animals required to achieve statistical significance (*p* < 0.05) with 80% power was met. A minimum of five animals per group was necessary based on a large effect size (partial η^2^ >  0.14) and test power exceeding 80% for the treatment effect. The experiment followed a completely randomized design. Mice were randomly assigned to one of four groups (n = 6 animals/group): sham-operated group treated with saline (Sham), ovariectomized group treated with saline (OVX), sham-operated group treated with HA (Sham + HA), and ovariectomized group treated with HA (OVX + HA). HA was administered at a dose of 80 mg/kg daily for 28 days, starting 2 weeks after surgical recovery. This dosage and treatment duration were determined based on previous studies conducted by our group using a periodontitis model [17,29,32]. At the end of the experiment, the animals were euthanized, and the uterus, femur, gastrocnemius muscle, liver, and kidneys were collected.

### 2.4. Protocol of Sham and Ovariectomy (OVX)

After a two-week acclimation period, the animals were divided into two groups: one group underwent ovariectomy (OVX, n = 12) and the other received a sham operation (Sham, n = 12). The distribution of animals between groups was randomized, and group allocation was balanced based on body weight to ensure no significant differences in initial weight between the groups. In the OVX group, both ovaries were surgically removed, as described in a previous study [33]. The same procedure was performed in the sham-operated group, but the ovaries were left intact. All procedures were carried out under anesthesia, with ketamine (100 mg/kg) and xylazine (10 mg/kg).

### 2.5. Administration of Humic Acids (HA)

Two weeks after the surgical procedures, the animals were randomly assigned to four experimental groups based on the treatment (n = 6/group): sham-operated group treated with saline (Sham), ovariectomized group treated with saline (OVX), sham-operated group treated with HA (Sham + HA), and ovariectomized group treated with HA (OVX + HA). The HA solutions were prepared daily by dissolving the compound in saline solution. HA was administered daily by gavage for 28 days. In the Sham and OVX groups, 0.3 mL of saline solution was administered daily. At the end of the experimental period, the animals were weighed, anesthetized with intraperitoneal injections of xylazine hydrochloride (10 mg/kg) and ketamine hydrochloride (80 mg/kg), and then euthanized via cardiac puncture. After euthanasia, the uterus of the mice was weighed using an analytical balance.

### 2.6. Assessment of Bone Elementary Composition and Topography Using Scanning Electron Microscopy Coupled with Energy Dispersive X-Ray Spectroscopy (SEM/EDS)

The left femurs were immersed in hydrogen peroxide for 12 h to facilitate the removal of the remaining soft tissues and were subsequently dried at room temperature for two weeks. To evaluate the bone microstructure and identify any morphological characteristics in the bone, each sample was placed on the surface of an aluminum support using double-sided carbon tape and analyzed via scanning electron microscopy (SEM) (Vega 3 LMU, TESCAN, Brno-Kohoutovice, Czech Republic). Assessments were conducted on the distal femoral epiphysis, with images obtained at magnifications of 27× and 500×. The positioning of the samples in the equipment and the image acquisition were consistently performed by the same researcher (FGF). We evaluated the bone topography in the images captured at 500× magnification, focusing on aspects such as porosity, irregularities, and roughness, as previously described [34].

Additionally, we evaluated the elemental composition of the bone surface, including calcium, phosphorus, and carbon, using energy dispersive X-ray spectroscopy (EDS) with an X-MaxN detector (Oxford Instruments, Abingdon, United Kingdom). Spectra were acquired at an acceleration voltage of 20 kV and a working distance of 13 mm, with data analysis conducted using AZtec 3.1 software (Oxford Instruments, High Wycombe, UK) [34]. We also calculated the Ca/P, C/Ca, and C/P ratios, as described in a previous study [35].

### 2.7. Evaluation of the Redox Status

After euthanasia, fragments of the liver, kidney, and left gastrocnemius muscle were collected, immediately frozen in liquid nitrogen, and stored at −80 °C for the evaluation of oxidative stress markers. Each tissue sample was individually weighed and homogenized using a homogenizer (Ultra-turrax) at a ratio of 100 mg of tissue per 1 mL of KH_2_PO_4_ + K_2_HPO_4_ buffer. The resulting homogenate was centrifuged at 12,000 rpm for 10 min, and the supernatant from each sample was carefully collected, aliquoted, and stored at −80 °C. Protein concentrations in the homogenate were quantified using the Bradford method [36]. The concentration of hydroperoxide (H_2_O_2_) was determined following the protocol previously described [37]. Superoxide dismutase (SOD) activity was assessed based on the enzyme’s ability to inhibit the superoxide anion (O_2_^−^), thereby reducing the rate of pyrogallol auto-oxidation [38]. Catalase (CAT) activity was measured by the decomposition of hydrogen peroxide (H_2_O_2_) over one minute, with absorbance readings taken at a wavelength of 240 nm [39].

### 2.8. Statistical Analyses

The data underwent analysis of variance (ANOVA) with the F-test in a 2 × 2 factorial model (considering HA treatment and OVX as factors) to compare groups and interaction (*p* < 0.05). Bonferroni’s post hoc test was conducted when the F values indicated significant interaction (*p* < 0.05). GraphPad Prism version 8.00 (v. 8, GraphPad Software, San Diego, CA, USA) was utilized for data analysis.

## 3. Results

### 3.1. Uterine and Body Weight

**Figure 1:**
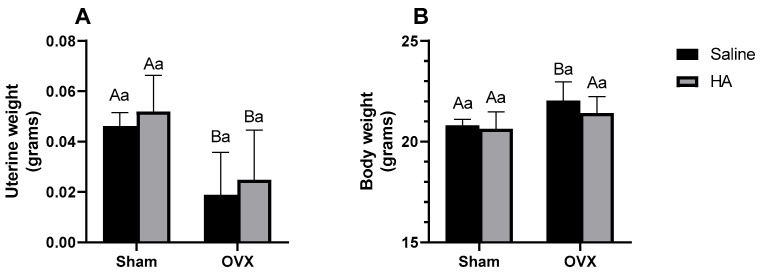
Uterine (**A**) and body weight (**B**) of ovariectomized (OVX) mice treated with humic acids (HA). ^A,B^ Uppercase letters denote significant differences between OVX and sham-operated groups (*p* < 0.05). ^a^ Lowercase letters indicate significant differences between groups treated and untreated with HA (*p* < 0.05). Data were analyzed using two-way ANOVA followed by Bonferroni post hoc test (n = 6 per group).

Animals in both OVX groups exhibited lower uterine weights compared to those in the Sham groups (between Sham and OXV group *p* = 0.024; between Sham + HA and OVX + HA *p* = 0.025—Figure 1A). Conversely, body mass was higher in OVX animals compared to Sham (*p* = 0.047—Figure 1B). No significant differences were observed in body mass between the OVX + HA group and either the OVX or Sham + HA groups (*p* > 0.05).

### 3.2. Elemental Composition and Topography of Femur Assessed by SEM/EDS

**Figure 2:**
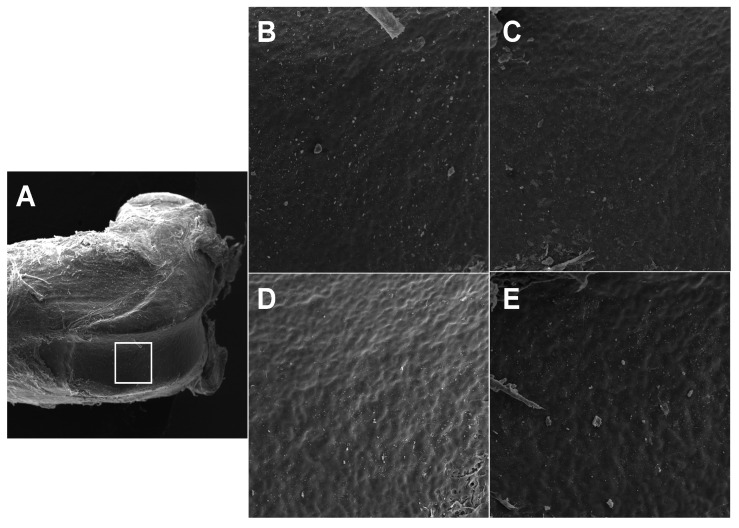
Representative images showing the topography of the distal femoral epiphysis in ovariectomized (OVX) mice treated with humic acids (HA). Magnification: (**A**)—27×; (**B**–**E**)—500×, obtained via scanning electron microscopy (SEM). (**A**): The square highlights the standardized region selected for topographical evaluation. (**B**): Sham-operated group treated with saline (Sham). (**C**): Sham-operated group treated with HA (Sham + HA). (**D**): Ovariectomized group treated with saline (OVX). (**E**): Ovariectomized group treated with HA (OVX + HA).

**Figure 3:**
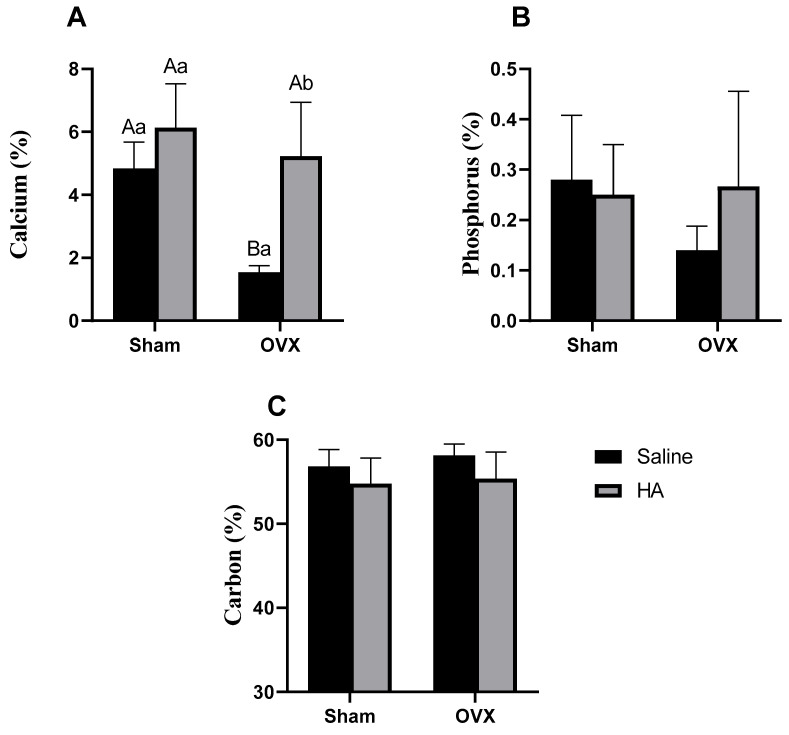
Elemental composition of the femoral epiphysis surface in ovariectomized (OVX) mice treated with humic acids (HA). (**A**): Calcium percentage in distal femoral epiphysis. (**B**): Phosphorus percentage in distal femoral epiphysis. (**C**): Carbon percentage in distal femoral epiphysis. ^A,B^ Uppercase letters denote significant differences between OVX and sham-operated groups (*p* < 0.05). ^a,b^ Lowercase letters indicate significant differences between groups treated and untreated with HA (*p* < 0.05). Data were analyzed using two-way ANOVA followed by Bonferroni post hoc test (n = 6 per group).

**Figure 4:**
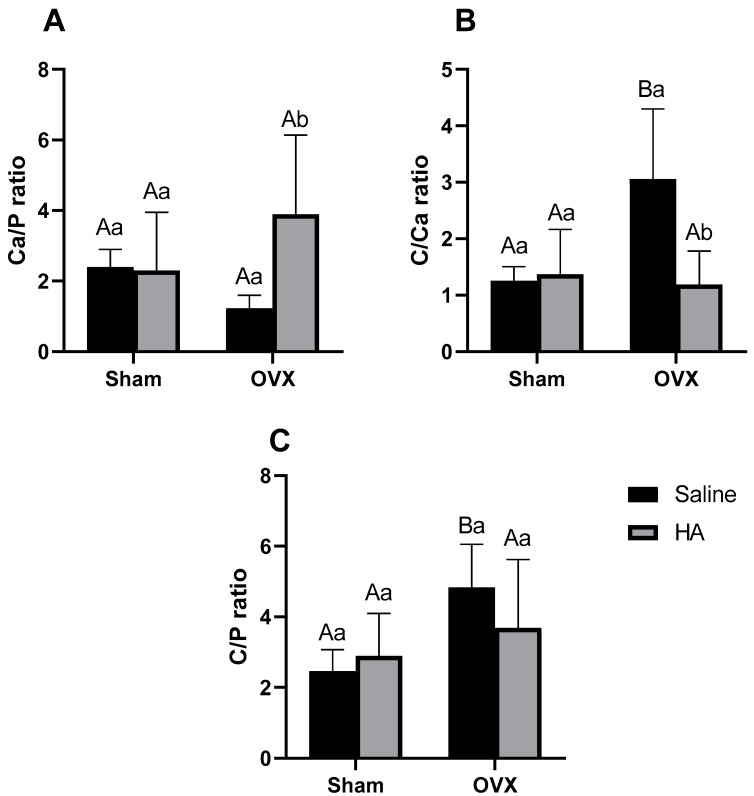
Ca/P (**A**), C/Ca (**B**), and C/P (**C**) ratios of the femoral epiphysis surface in ovariectomized (OVX) mice treated with humic acids. (HA). ^A,B^ Uppercase letters denote significant differences between OVX and sham-operated groups (*p* < 0.05). ^a,b^ Lowercase letters indicate significant differences between groups treated and untreated with HA (*p* < 0.05). Data were analyzed using two-way ANOVA followed by Bonferroni post hoc test (n = 6 per group).

No significant differences were observed in the topography (porosity, irregularities, and roughness) of the distal femoral epiphysis among the experimental groups (Figure 2A–E). However, elemental composition analysis revealed that calcium percentages were lower in the OVX group compared to the Sham group (*p* = 0.000—Figure 3A). Additionally, calcium levels were higher in the OVX + HA group compared to the OVX group (*p* = 0.000—Figure 3A). No significant differences were found in phosphorus (effect of ovariectomy *p* = 0.361; effect of HA treatment *p* = 0.240—Figure 3B) and carbon levels (effect of ovariectomy *p* = 0.743; effect of HA treatment *p* = 0.362—Figure 3C). The Ca/P ratio did not differ significantly between the Sham and OVX groups (between Sham and OXV group *p* = 0.502; between Sham + HA and OVX + HA *p* = 0.247); however, OVX animals showed lower values for this parameter compared to the OVX + HA group (*p* = 0.020; Figure 4A). The C/Ca ratio was higher in the OVX group compared to both the Sham and OVX + HA groups (*p* < 0.003; Figure 4B). Lastly, the C/P ratio was elevated in the OVX group compared to the Sham group (*p* < 0.028), with no significant differences observed following HA treatment (*p* > 0.461; Figure 4C).

### 3.3. Oxidative Stress Markers in the Gastrocnemius Muscle

**Figure 5:**
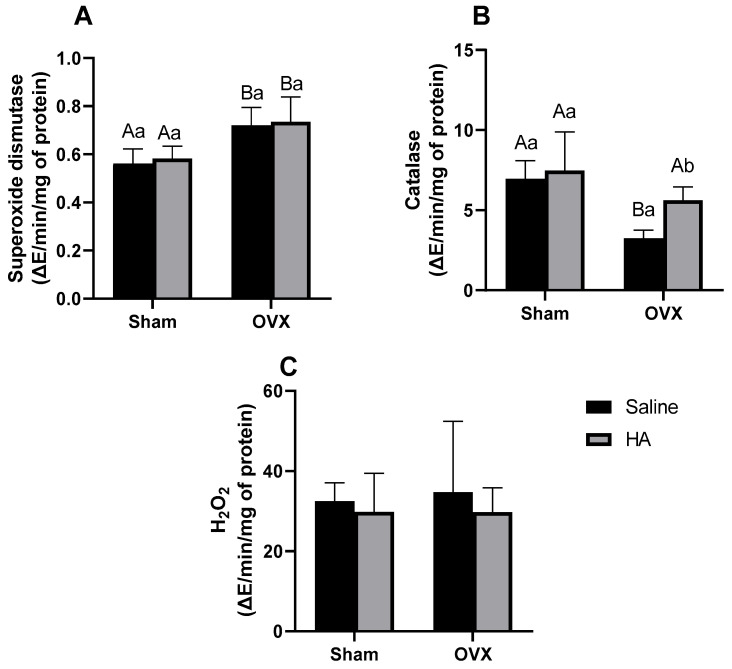
Oxidative stress markers in the gastrocnemius muscle of ovariectomized (OVX) mice treated with humic acids (HA). (**A**): Superoxide dismutase (**B**): Catalase. (**C**): H_2_O_2_. ^A,B^ Uppercase letters denote significant differences between OVX and sham-operated groups (*p* < 0.05). ^a,b^ Lowercase letters indicate significant differences between groups treated and untreated with HA (*p* < 0.05). Data were analyzed using two-way ANOVA followed by Bonferroni’s post hoc test (n = 6 per group).

SOD activity levels in the gastrocnemius muscle were higher in both OVX groups compared to the Sham groups (between Sham and OXV group *p* = 0.007; between Sham + HA and OVX + HA *p* = 0.010—Figure 5A), with no significant effect observed from HA treatment. Conversely, CAT activity was lower in OVX animals compared to the Sham group (*p* < 0.001; Figure 5B); however, CAT activity was significantly increased in OVX groups treated with HA (*p* < 0.040; Figure 5B). No significant differences were observed in H_2_O_2_ activity among the groups (effect of ovariectomy *p* = 0.361; effect of HA treatment *p* = 0.240—Figure 5C).

### 3.4. Oxidative Stress Markers in the Liver

**Figure 6:**
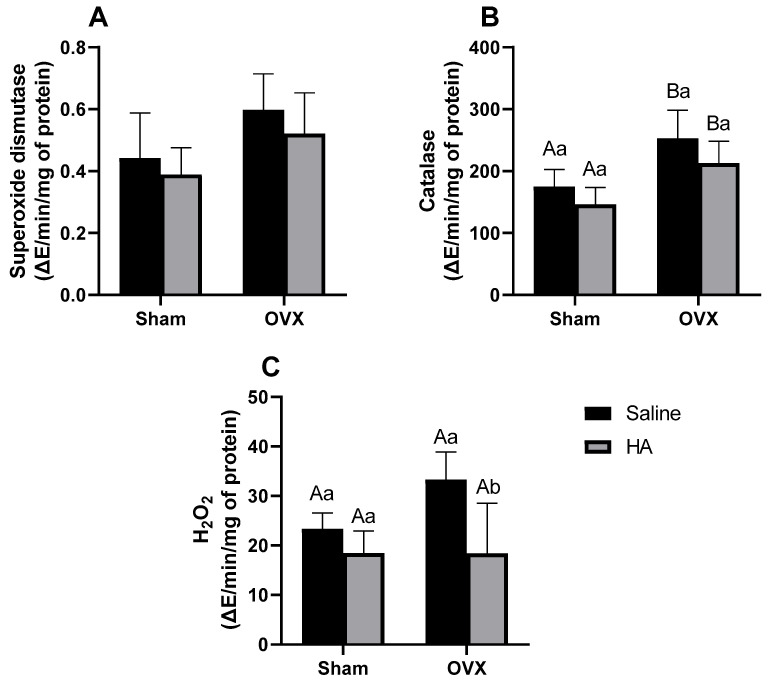
Oxidative stress markers in the liver of ovariectomized (OVX) mice treated with humic acids (HA). (**A**): Superoxide dismutase (**B**): Catalase. (**C**): H_2_O_2_. ^A,B^ Uppercase letters denote significant differences between OVX and sham-operated groups (*p* < 0.05). ^a,b^ Lowercase letters indicate significant differences between groups treated and untreated with HA (*p* < 0.05). Data were analyzed using two-way ANOVA followed by Bonferroni’s post hoc test (n = 6 per group).

No significant differences were observed in hepatic SOD activity among the groups (effect of ovariectomy *p* = 0.361; effect of HA treatment *p* = 0.240—Figure 6A). Hepatic CAT activity was higher in OVX animals compared to both the Sham (*p* = 0.005) and OVX + HA groups (*p* = 0.018; Figure 6B). H_2_O_2_ levels were reduced in the OVX + HA group compared to the untreated OVX animals (*p* < 0.003; Figure 6C), with no significant differences observed between the other groups.

### 3.5. Oxidative Stress Markers in the Kidney

**Figure 7:**
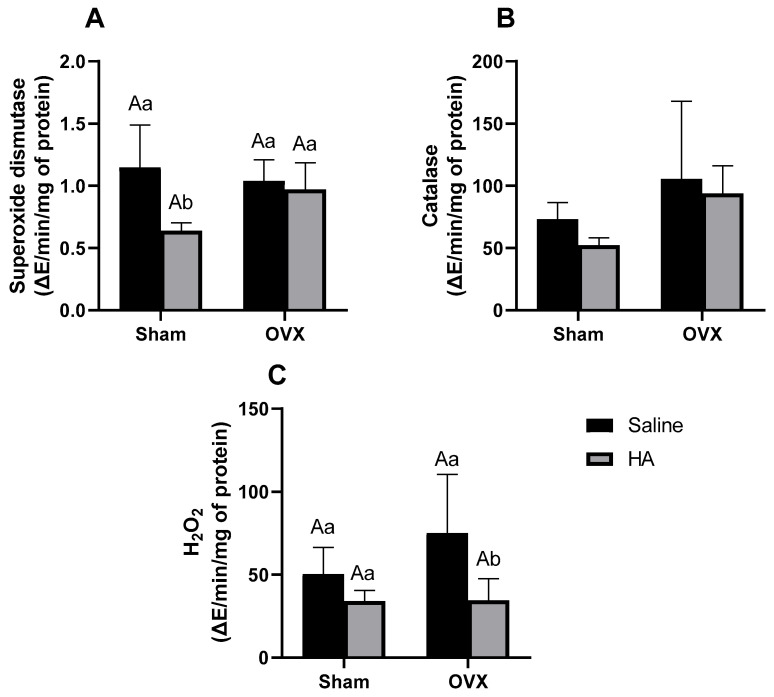
Oxidative stress markers in the kidney of ovariectomized (OVX) mice treated with Humic Acids (HA). (**A**): Superoxide dismutase (**B**): Catalase. (**C**): H_2_O_2_. ^A^ Uppercase letters denote significant differences between OVX and sham-operated groups (*p* < 0.05). ^a,b^ Lowercase letters indicate significant differences between groups treated and untreated with HA (*p* < 0.05). Data were analyzed using two-way ANOVA followed by Bonferroni’s post hoc test (n = 6 per group).

SOD activity in the kidneys was reduced in the Sham group treated with HA compared to the untreated Sham group (*p* = 0.003; Figure 7A), with no significant differences observed between the other groups. Renal CAT activity did not differ across treatments (effect of ovariectomy *p* = 0.739; effect of HA treatment *p* = 0.253—Figure 7B). H_2_O_2_ levels were lower in the OVX group treated with HA compared to the untreated OVX group (*p* = 0.014; Figure 7C), with no significant differences observed among the other groups.

## 4. Discussion

The main findings of our study were the improvement in bone elemental composition in ovariectomized animals treated with HA compared to those untreated. Additionally, we observed that in mice induced to the menopause model, treatment with HA increased CAT activity in the gastrocnemius muscle and reduced H_2_O_2_ levels in both the liver and kidneys compared to untreated animals. To our knowledge, this is the first study to evaluate the effects of HA treatment in an experimental menopause model. Therefore, our pioneering findings support previous results linking the protective effects of these agents on bone [17,25,27,28,29] and their modulation of oxidative stress markers [40,41].

Ovariectomy is a surgical procedure used to simulate the cessation of gonadal estrogen production similar to that which occurs after menopause [42]. The absence of these hormones leads to hallmark outcomes of this condition, including reduced uterus mass and increased body weight [43]. In fact, in our study, the uterine weight of animals in both OVX groups was lower than that of the Sham groups, confirming the effectiveness of the surgery. Additionally, we observed that OVX animals had a higher body weight than the Sham group at the end of the experimental period. The increase in body mass in response to reduced circulating estrogen levels occurs because these hormones play a key role in regulating energy metabolism and appetite [44]. Furthermore, the absence of estrogens decreases the basal metabolic rate, favoring energy accumulation in body fat [45,46].

Other classic outcomes of ovariectomy include a reduction in bone mineral density [47] and alterations in oxidative stress within metabolically active tissues [48]. In our study, we observed that calcium levels in the femur of OVX animals were approximately 60% lower compared to those in the Sham group, which was statistically significant. Additionally, femoral phosphorus levels in OVX animals were about 50% lower than in the Sham group; however, this difference was not statistically significant. The deposition of minerals such as calcium and phosphorus in bone is mediated by estrogen, which inhibits osteoclast activity and, consequently, enhances osteoblast activity [49]. Therefore, with reduced levels of these hormones, osteoclast activity increases, promoting bone resorption and reducing the mineral matrix [50]. Regarding the oxidative profile, estrogen generally exerts an antioxidant effect, and its reduction can potentially increase oxidative damage in various tissues [48]. This was partially observed in our study, as evidenced by the increased H_2_O_2_ levels in the liver and kidney tissues of OVX animals compared to the Sham group.

Considering the detrimental effects caused by decreased estrogen levels in postmenopause, various strategies have been employed to mitigate damage to bone tissue and oxidative stress [14]. Among these agents, HA has emerged as a promising candidate due to its antioxidant properties [32] and potential to improve bone composition [17,29,51]. In our study, we observed that ovariectomized animals treated with HA exhibited higher calcium levels and an increased Ca/P ratio compared to untreated animals, with values comparable to those of the Sham group. An elevated Ca/P ratio indicates greater cortical mineralization [52]. Healthy cortical bone is characterized by slow turnover and low porosity, with its minerals being less susceptible to ionic substitution compared to trabecular bone [53]. Thus, cortical bone loss is associated with trabecularization of the endocortical surface and increased cortical porosity [53].

Another noteworthy finding in our study was the reduction in the C/Ca and C/P ratios in OVX animals treated with HA compared to untreated ones. Elevated C/Ca and C/P ratios indicate a higher protein-to-mineral content in the bone matrix [54]. Conversely, decreasing these ratios may indicate enhanced bone mineralization [35]. In previous studies conducted by our group, we observed that the administration of HA in an experimental periodontitis model increased the Ca and P content in the mandibular alveolar bone of the animals [17,29]. The mechanisms through which HA confers protection against bone demineralization remain unclear. However, these compounds are believed to enhance intestinal absorption and mineral bioavailability [55], thereby providing more substrate for deposition in the bone [51]. Furthermore, the anti-inflammatory properties of these agents may inhibit osteoclast activation, reducing bone resorption [28]. Nonetheless, further studies are needed to elucidate the mechanisms through which HA preserves the bone mineral matrix.

Regarding the oxidative stress markers evaluated in the present study, we observed that ovariectomy altered SOD and CAT levels in the gastrocnemius muscle and increased CAT activity in the liver compared to Sham animals. This increase in CAT activity in the liver may be linked to a compensatory mechanism of this enzyme to combat excess free radicals caused by reduced estrogen levels [56]. Indeed, menopause has been reported to impair antioxidant activity [57]. Estrogen exerts an antioxidant effect, and its reduction can potentially enhance oxidative damage in various tissues [48]. This was observed in our study, as indicated by the trend toward higher H_2_O_2_ levels in the hepatic and renal tissues of OVX animals compared to the Sham group. However, although no significant differences in enzymatic activity were observed in the liver between Sham and OVX animals, H_2_O_2_ levels were reduced in OVX animals treated with HA. This protective effect of HA may be attributed to their structural characteristics, particularly the presence of quinone-like structures that enable the molecule to transfer electrons independently of mitochondrial enzymes, thereby directly neutralizing free radicals [58]. Additionally, oxidized phenolic groups that form phenoxyl radicals may also contribute to the antioxidant activity of HA [59].

HA has the potential to reduce oxidative damage by disrupting the radical chain reaction, thereby preventing harm to cell membranes and biological macromolecules. In a previous study conducted by our group, we characterized HA extracted from biomass vermicompost and identified the presence of 32% aromatic hydrogens, which play a crucial role in the antioxidant activity of this compound [32]. The quinone-like structures in HA facilitate electron acceptance and transfer, enabling the reduction of hydrogen peroxide without mitochondrial enzyme involvement [60]. Additionally, it was demonstrated that the balance between electron acceptors (quinones) and donors (phenols) varies depending on the origin of HA, confirming that phenolic groups slow the oxidative transformation of quinones [61]. Furthermore, due to its polyanionic nature, HA can bind metal ions through various chemical and physical mechanisms [60]. This chelating ability allows HA to sequester pro-oxidant metal ions, which would otherwise catalyze the formation of ROS via Fenton reactions [60]. By binding these metals, these compounds reduce their availability for oxidative processes, thereby mitigating oxidative stress [62]. It is important to emphasize that the origin of HA may impart distinct functional characteristics [63,64], necessitating an investigation into the molecular mechanisms underlying the modulation of oxidative stress markers. In the present study, we did not evaluate these molecular mechanisms. This limitation should be overcome in future studies through RNA sequencing or proteomic analyses to investigate the underlying causes of this modulation.

Despite the well-documented beneficial effects of HA and their classification as safe compounds in the literature [55], we observed a decrease in SOD activity in the kidneys of Sham + HA animals compared to Sham animals. SOD is an antioxidant marker with the specific ability to scavenge intracellular and extracellular superoxide radicals in vivo by decomposing them into hydrogen peroxide and oxygen molecules [65]. Therefore, although H_2_O_2_ levels showed no significant differences between the Sham and Sham + HA groups, and these markers improved in OVX animals treated with HA, it is crucial for future studies to explore the specific responses of renal tissue to treatment with these compounds.

Although most of the literature highlights the potential therapeutic effects of HA in human cell culture studies [58,66] and preclinical research [17,32,67], the oral administration of a commercial formulation of these compounds has already been investigated for its effects on the gut microbiota of healthy individuals, demonstrating potential in modulating the innate colonic microbiome [68]. Additionally, in a double-blind, placebo-controlled study, the administration of brown coal-derived potassium humate (a product with high levels of HA) showed potential as an anti-inflammatory agent in treating atopic conditions such as allergic rhinitis [69]. Furthermore, in a randomized, double-blind, placebo-controlled trial, the administration of Shilajit extract (a compound containing 60–80% humic substances) was found to attenuate increased bone turnover, inflammation, and oxidative stress in women with osteopenia [70]. However, to date, no clinical or preclinical studies have investigated the effects of isolated HA on osteopenia or osteoporosis, making our study the first to explore this relationship.

Despite the careful execution of the experiment and analyses, our study is not without limitations. In this study, we did not evaluate markers of bone remodeling such as RANKL (Receptor Activator of Nuclear Factor Kappa-B Ligand) and OPG (osteoprotegerin), as well as components of the bone cellular matrix (osteoclasts, osteoblasts, and osteocytes) that are altered in osteoporosis. Another limitation of this study is that we did not conduct experiments to assess the molecular mechanisms involved in the modulation of oxidative stress markers. Therefore, future studies should consider evaluating these parameters to determine the influence of HA on these specific parameters. Additionally, the present study examined the effects of only 28 days of treatment. Given that the changes induced by ovariectomy are permanent and irreversible without treatment, it would be valuable for future studies to assess the effects of long-term HA administration. Despite the outlined limitations, the design and findings of this study are novel, original, and promising. Our results provide a strong foundation for future clinical research on the use of HA derived from biomass vermicomposting to prevent and treat conditions associated with estrogen decline during climacteric and menopause. Unlike conventional sources, this sustainable approach offers a potentially more bioavailable and environmentally friendly alternative, reducing reliance on chemically synthesized humic acid. Furthermore, our study pioneers the application of biomass-derived humic acid in a preclinical model of estrogen deficiency, addressing a critical gap in the current literature and presenting a viable strategy for both health and environmental sustainability.

## 5. Conclusions

Treatment with HA derived from agricultural biomass vermicompost improves the elemental composition of bone and modulates oxidative stress markers in the gastrocnemius muscle, liver, and kidneys of ovariectomized animals.

## Data Availability

The raw data supporting the conclusions of this article will be made available by the authors on request.
